# REST-dependent downregulation of von Hippel-Lindau tumor suppressor promotes autophagy in SHH-medulloblastoma

**DOI:** 10.1038/s41598-024-63371-7

**Published:** 2024-06-12

**Authors:** Ashutosh Singh, Donghang Cheng, Jyothishmathi Swaminathan, Yanwen Yang, Yan Zheng, Nancy Gordon, Vidya Gopalakrishnan

**Affiliations:** 1 Department of Pediatrics Research, The University of Texas MD Anderson Cancer Center, 1515 Holcombe Blvd, Unit 853, Houston, TX 77030 USA; 2 Center for Cancer Epigenetics, The University of Texas MD Anderson Cancer Center, 1515 Holcombe Blvd, Houston, TX 77030 USA; 3 Brain Tumor Center, The University of Texas MD Anderson Cancer Center, 1515 Holcombe Blvd, Houston, TX 77030 USA; 4 The University of Texas MD Anderson Cancer Center and UTHealth Graduate School for Biomedical Sciences, 6767 Bertner Ave, S3.8344 Mitchell BSRB, Houston, TX 77030 USA

**Keywords:** REST, Von Hippel-Lindau (VHL), Hypoxia-inducible factor 1-alpha (HIF1α), Ubiquitination, Autophagy, Medulloblastoma, Paediatric cancer, Cancer genetics

## Abstract

The *RE1* silencing transcription factor (REST) is a driver of sonic hedgehog (SHH) medulloblastoma genesis. Our previous studies showed that REST enhances cell proliferation, metastasis and vascular growth and blocks neuronal differentiation to drive progression of SHH medulloblastoma tumors. Here, we demonstrate that REST promotes autophagy, a pathway that is found to be significantly enriched in human medulloblastoma tumors relative to normal cerebella. In SHH medulloblastoma tumor xenografts, REST elevation is strongly correlated with increased expression of the hypoxia-inducible factor 1-alpha (HIF1α)—a positive regulator of autophagy, and with reduced expression of the von Hippel-Lindau (VHL) tumor suppressor protein – a component of an E3 ligase complex that ubiquitinates HIF1α. Human SHH-medulloblastoma tumors with higher REST expression exhibit nuclear localization of HIF1α, in contrast to its cytoplasmic localization in low-REST tumors. In vitro, REST knockdown promotes an increase in VHL levels and a decrease in cytoplasmic HIF1α protein levels, and autophagy flux. In contrast, REST elevation causes a decline in VHL levels, as well as its interaction with HIF1α, resulting in a reduction in HIF1α ubiquitination and an increase in autophagy flux. These data suggest that REST elevation promotes autophagy in SHH medulloblastoma cells by modulating HIF1α ubiquitination and stability in a VHL-dependent manner. Thus, our study is one of the first to connect VHL to REST-dependent control of autophagy in a subset of medulloblastomas.

## Introduction

Medulloblastoma (MB) is an embryonal neuroepithelial tumor that accounts for 20% of all pediatric brain tumors and 10% of all childhood cancer deaths^1,2^. It is the most common malignant brain tumor in children, with nearly 500 new diagnoses each year in USA^3^. Approximately 20–30% of patients exhibit metastasis at the time of first diagnosis^4^. Despite significant advances in molecular stratification and understanding of MB biology, standard of care continues to involve surgical resection, craniospinal radiation and chemotherapy, which are known to cause quality of life issues^4,5^. On average, the 5-year survival is around 66%, with the primary determinant being the molecular subgroup of the tumors—Wingless (Wnt), Sonic Hedgehog (SHH), group 3, or group 4^1,5,6^.

The overall goal of our work is to understand the role of the *RE1* Silencing Transcription Factor (REST), a transcriptional repressor and canonical regulator of neurogenesis^7,8^, in SHH-MB tumorigenesis. In previous studies, we showed that REST expression is significantly elevated in the SHH-α and -β subtypes and is correlated with metastasis and poor survival^9^. REST is a regulator of stemness in cancer cells^10^. While REST knockdown is known to reduce the tumorigenic potential of MB cells, its ectopic expression in immortalized neural stem cells promotes tumor progression^9,11,12^. Studies with genetically engineered mice also revealed that while REST elevation alone is insufficient for MB development, it drives leptomeningeal dissemination in the context of constitutive activation of SHH signaling^9,13^.

In the current study, we demonstrate a role for REST in the regulation of autophagy in SHH-MB cells. Autophagy is a cellular process that tags misfolded proteins and damaged organelles for lysosome-mediated degradation and allows recovery of raw materials into the cytoplasm^14,15^. It is a cytoprotective mechanism in brain cells and serves to prevent the accumulation of damaged intracellular components, thereby facilitating cellular homeostasis and ensuring cell survival under conditions of stress and nutrient starvation^16,17^. Thus, it is essential for brain health^17^. Interestingly, REST function is important during aging and in the pathology of neurodegenerative disorders, and appears to involve dysregulation of autophagy^18,19^. However, REST upregulation which accompanies exposure of neurons to high glucose and palmitic acid, is also associated with a decrease in autophagy and an increase in senescence-like phenotypes^20^. Other studies have also shown that REST downregulation results in a failure of autophagy, loss of proteostasis, increased oxidative stress, senescence, and cell death in mouse neurons^21^. Thus, REST's role in autophagy seems to be cell-context and disease-specific.

As in neuronal homeostasis, autophagy has seemingly opposite roles in cancer, where it can contribute to, or block tumor progression in neural cancers such as atypical teratoid/rhabdoid tumor, low-grade glioma, diffuse intrinsic pontine glioma, ependymoma^22–26^. It is also implicated in therapy resistance and in tumor recurrence^27,28^. Surprisingly, only a few studies have investigated autophagy in the context of MBs. In one such study, miR-30a and miR-204 were shown to regulate autophagy and affect the growth of MB cells in vitro and their tumorgenicity in vivo^29,30^. Hypoxia, which is observed in pediatric brain tumor samples, is a potent inducer of autophagy. Consistent with this, inhibition of hypoxia-inducible factor- α (HIF1α) – a key regulator of autophagy, reduced MB cell proliferation through epigenetic silencing of ATG16L1 protein, which promotes autophagosome biosynthesis^31^. A mass spectrometry-based multi-omics pilot study of cerebrospinal fluid from recurrent MB patients also demonstrated that under hypoxic conditions, the activation of autophagy triggers drug resistance of stem-like MB cells^32^. The data described in the current work suggests that REST-dependent decline in the levels of the von Hippel Lindau (VHL) tumor suppressor protein—a HIFα-specific E3 ubiquitin ligase, promotes stabilization and nuclear localization of HIF1α to drive autophagy in SHH-MB cells.

## Materials and methods

### Gene expression profile in patient samples

The gene expression profile of tumors from MB patients was analyzed using previously published publicly available RNAseq (GSE148389) and microarray (GSE202043, GSE124814, and GSE85217) datasets. Differential expression of genes and unsupervised clustering analysis was performed using the R2: Genomics Analysis and Visualization Platform (http://r2.amc.nl). A value of p < 0.05 was considered significant. Gene ontology p-values were not corrected for multiple testing.

### Cell culture

Two SHH-MB cell lines, Daoy and UW228 were used here due to differences in REST expression. Daoy cells were purchased from the American Type Culture Collection (Manassas). UW228 cells were a kind gift from Dr. John Silber at the University of Washington. Both cell lines were maintained in Dulbecco's modified Eagle's medium (Sigma), supplemented with 10% fetal bovine serum (Sigma Aldrich), 1% Antibiotic–Antimycotic (Thermo Fisher Scientific), and 1% sodium pyruvate (Thermo Fisher Scientific) and grown in a humid environment at 37 °C with 5% CO2.

### Patient-derived xenograft models

Patient-derived xenograft (PDX) models (RCMB-018, -024 and -054) were a kind gift of Dr. Robert Wechsler-Reya at Columbia University. Serial transplantation of tumors was carried out in NOD.Cg-*Prkdc*^*scid*^* Il2rg*^*tm1Wjl*^/SzJ (NSG) mice (Jackson Laboratory, Bar Harbor, ME), by intracranial inoculation of tumor cells using a stereotactic device as described previously^9^. Housing, maintenance and experiments involving mice were done in accordance with and following approval by The University of Texas MD Anderson Cancer Center’s Institutional Animal Care and Use Committee (IACUC). Our study is reported in accordance with ARRIVE guidelines (https://arriveguidelines.org).

### Immunohistochemistry (IHC)

Mouse brain tissues were fixed in 10% buffered formalin phosphate for 48 hours and embedded in paraffin. Eight-micrometer-thick brain sections were used for IHC analysis. Sections were deparaffinized with heat and xylene and rehydrated with ethanol and water. Antigen retrieval was performed in citrate antigen retrieval buffer (pH 6) for 30 minutes at 95 ºC in the PT module (ThermoFisher). Sections were washed with 0.1% Tween-20 in PBS (PBST) and then treated with 3% H_2_O_2_ solution for 10 min to block endogenous peroxidase. Nonspecific binding of rabbit and mouse antibodies was blocked with 1% normal goat serum and, a solution provided in the Mouse-on-Mouse kit (MOM kit, Vector labs), respectively. Sections were then incubated with primary antibodies as indicated below in blocking buffer at 4 °C overnight. Primary antibody was detected using a biotinylated secondary antibody provided with the ABC or MOM kit and then incubated with streptavidin-HRP (ABC kit, Vector labs) according to the manufacturers’ instructions. All incubations were performed under humidified conditions. Slides were washed with TBS-T, developed using the DAB kit (Vector labs), and counterstained with hematoxylin. After dehydration and mounting, slides were visualized under a Nikon ECLIPSE E200 microscope, and images were captured under 4x, 10x, and 40x magnification using an Olympus SC100 camera. Analyses were performed using Olympus cellSens Entry software. The following antibodies were used: rabbit anti-REST, 1:200 (Abcam, Cat no# ab202962); rabbit anti-LC3B, 1:100 (Novus Biologicals, Cat no# NB600); rabbit anti-p62, 1:400 (Cell Signaling Technology, Cat no# 88588); rabbit anti- HIF1α, 1:100 (Novus Biologicals, Cat no# NB100-134) and rabbit anti-VHL, 1:200 (ThermoFisher Scientific, Cat no# PA5-27322).

### Lentiviral infection

Embryonic kidney (HEK) 293 T cells were co-transfected with a lentiviral vector (Phage -ef1α-IRES-GFP) backbone or a construct expressing the gene of interest along with packaging plasmid (PAX2) and envelope plasmid (MD2). Lentiviral particles were collected 48 h post-transfection. Cells were transduced with the collected viral supernatant in the presence of Polybrene (8 μg/mL) and incubated for 48 h. Infected cells were then cultured in a medium containing 2 μg/mL puromycin for up to 1 week for selection.

### Western blot

Cells extracts were prepared for Western blot analysis by incubating the cells in lysis buffer (50 mM Tris–HCl pH 8.0, 50 mM NaCl, 1% NP-40, 0.5% sodium deoxycholate, 0.1% SDS, and protease/phosphatase inhibitors) for 30 min on ice. The lysates were clarified by centrifugation at 13,000×*g* for 10 min at 4C, and the supernatants were collected and boiled in Laemmli buffer (Bio-Rad). Proteins were separated by electrophoresis on 10% SDS–polyacrylamide gels, transferred to Hybond-P polyvinylidene difluoride (PVDF) membranes (GE Healthcare), and analyzed by Western blotting with the indicated primary antibodies and HRP-conjugated goat anti-mouse or anti-rabbit secondary antibodies (GE). Protein bands were developed using SuperSignal West Dura Extended Duration Substrate (Thermo Fisher Scientific) and detected using Kodak Medical X-Ray Processor 104 (Eastman Kodak Company) and ChemiDoc Touch Imaging System (Bio-Rad). Images were analyzed using Image Lab software version 5.2.1 (Bio-Rad). The following primary antibodies were used: rabbit anti-REST, 1:1000 (Sigma, Cat no# 07-579); rabbit anti-LC3B, 1:1000 (Novus Biologicals, Cat no# NB600); rabbit anti-p62, 1:1000 (Cell Signaling Technology, Cat no# 88588); rabbit anti- HIF1α, 1:1000 (Novus Biologicals, Cat no# NB100); rabbit anti-VHL, 1:1000 (ThermoFisher Scientific, Cat no# PA5-27322); rabbit anti-Histone H3, 1:1000 (ThermoFisher Scientific, Cat no# PA5-16183); mouse anti-Beta actin, 1:7000 (Sigma, Cat no# A1978).

### Drug studies

To evaluate the effect of Bafilomycin A1 (Cell Signaling Technology, Cat no# 54645S) and Hydroxychloroquine (Cell Signaling Technology, Cat no# 85523S) on MB viability, cells were seeded in a 96-well plate at a density of 5 × 10^3 cells per well, and then treated with vehicle [0.2% dimethyl sulfoxide (DMSO)] or different concentrations of the above drugs for 48 and 72 h. Cell viability was measured by MTT assays and absorbance measurements were carried out at 570 nm using the CLSRIOStar Plus Plate Reader.

### Immunofluorescence assays

Cells were grown on 22 × 22 mm coverslips. After achieving 70–80% confluency, cells were washed with phosphate-buffered saline (PBS) and fixed with 4% paraformaldehyde (PFA) for 5–7 min. Following multiple washes with PBS, cells were permeabilized with 0.5% Triton X-100 for 5–7 min and washed once again with PBS, followed by blocking for 1 h at room temperature using 1% bovine serum albumin (BSA). Next, primary antibodies (Anti-LC3B 1:1000—Cat no# NB600; Novus Biologicals) were added to the coverslips at dilutions stated below and incubated overnight at 4 °C in a moist and humid chamber. Cells were washed thrice with PBS and incubated with secondary antibodies (Thermofisher; Cat no# A-21246) for 1 h at room temperature. Stained cells were imaged using a Leica DMi8 fluorescence microscope.

### Immunogold labeling

For double-labeling experiments, cultured cells were fixed with a solution containing 0.1% glutaraldehyde plus 2% paraformaldehyde in 0.1 M PBS, pH 7.3, then washed with sterile water overnight at 4 °C. Next, samples were dehydrated with increasing concentrations of ethanol embedded in Lowicryl resin and subjected to UV polymerization for 2 days at − 20 °C followed by 3 days at room temperature. Post-embedding immunogold labeling was carried out on 70 nm ultrathin sections on formvar coated nickel grids to detect the presence of LC3B. These ultrathin sections were conditioned in Millipore-filtered block reagent consists of 0.05 M glycine and 2% BSA in 0.1 M PBS, pH 7.3 for a duration of 30 min at room temperature. Sections were then incubated overnight at 4 °C with a primary antibody against anti-LC3B (1:100, Novus Biologicals; Cat no# NB600) in blocking buffer. After rinsing with PBS, sections were incubated for 2 h at room temperature with a secondary antibody conjugated to gold particles (dilution: 1:20, Jackson ImmunoResearch, Cat no# 111-195-144) diluted to a ratio of 1:40 in blocking buffer. Samples were rinsed in PBS and fixed with 2.5% glutaraldehyde (v/v) in PBS for a period of 15 min and subjected to uranyl acetate and lead citrate for contrast staining. Electron micrographs were captured using a JEM 1010 transmission electron microscope (JEOL, USA, Inc., Peabody, MA) at an accelerating voltage of 80 kV. Digital images were obtained using the AMT Imaging System (Advanced Microscopy Techniques Corp, Danvers, MA).

### Co-immunoprecipitation

Daoy and UW228 cells were washed with ice-cold PBS and lysed in mild lysis buffer (50 mM Tris–HCl pH 7.5, 150 mM NaCl, 1 mM EDTA, 1% Triton X-100, 5 mM EDTA) with a protease inhibitor cocktail (Roche) and phosphatase inhibitor (Sigma) and sonicated. Lysates were incubated with mouse IgG (as a control), anti-HIF1α (Novus Biologicals; NB100-105) primary antibody overnight at 4 °C and then incubated with A/G ultralink resin (Thermo Fisher Scientific) for 1.5 h at 4 °C. After four washes with lysis buffer, beads were boiled in loading buffer, separated by SDS-PAGE, transferred onto PVDF membranes and analyzed by Western blotting using anti-HIF1α (Novus Biologicals; Cat no# NB100-134), anti-ubiquitin (Cell Signaling Technology; Cat no# 3933) and anti-VHL (Novus biologicals; NB100-41384) antibodies.

## Results

### Autophagy is a significantly enriched pathway in human MB samples

We had previously shown that REST elevation in human SHH MB cell lines upregulated HIF1α levels^13^. Elevation of HIF1α can contribute to cell proliferation and autophagy^33,34^, which prompted us to investigate if human MB samples exhibit differential enrichment of autophagy-related genes compared to normal cerebellum. To this end, we used the R2 genomics and visualization platform to study two different MB patient datasets (GSE202043 and GSE148389) and identify enriched pathways in MB patients compared to the normal cerebellum. As expected, pathways related to neurogenesis and neuron differentiation, brain development and cell cycle were enriched in MBs (Fig. S1a,b). Additionally, autophagy-related pathways were also significantly enriched in human MB tumors compared to normal cerebellum (Fig. S1a,b). These molecular pathways were found to be significantly enriched in both the datasets. Gene ontology information for these differentially enriched pathways, including autophagy, is shown in Supplemental data (Spreadsheet S1). Further, genes could be clearly separated as having high or low expression in normal samples, a distinction that was lost in human MB tumors (Fig. S2a). These observations were recapitulated in the GSE148389 dataset (Fig. S2b). The list of autophagy-related genes included for clustering analysis is provided in Spreadsheet S1.

**Figure 1:**
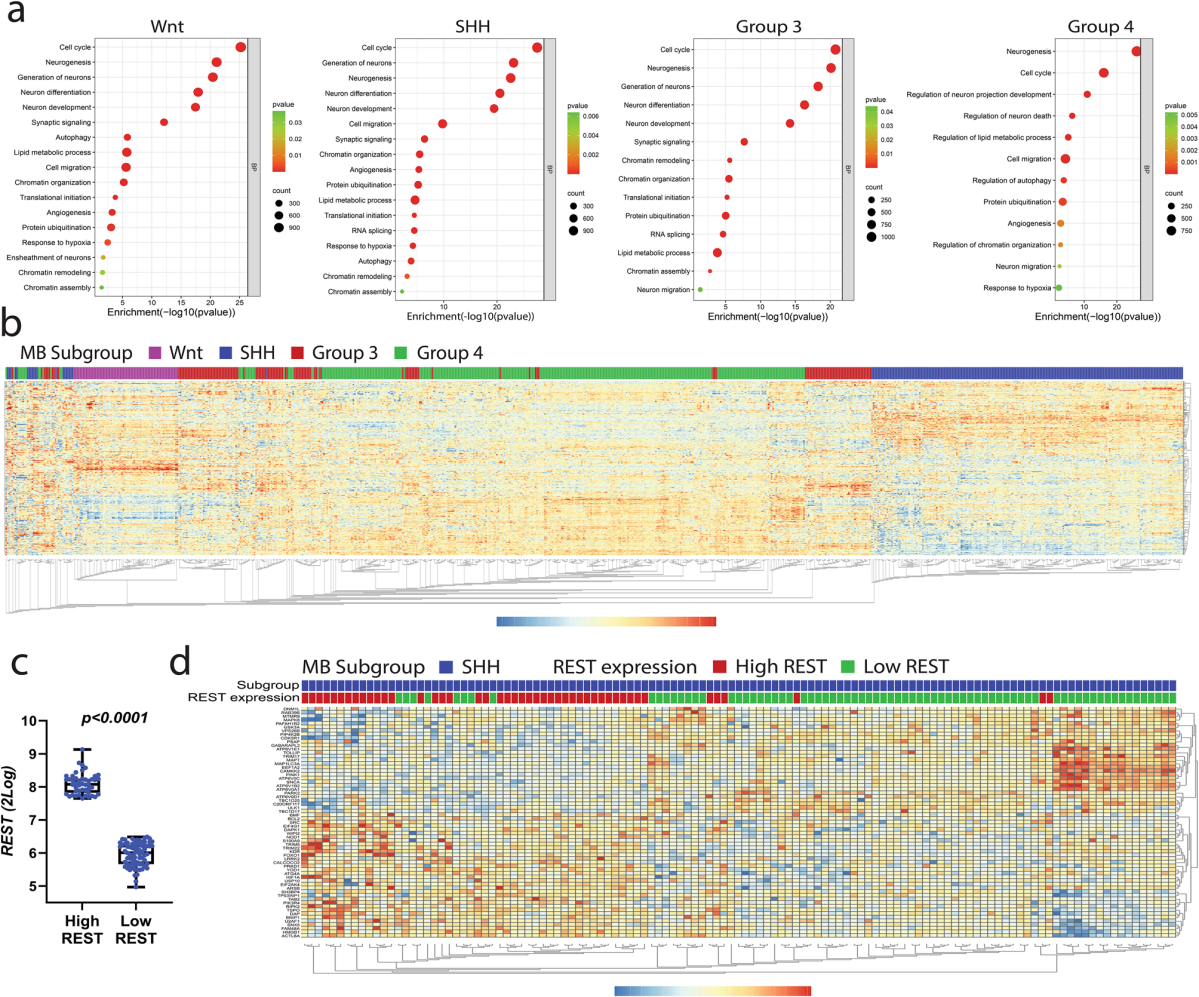
Autophagy is an enriched pathway in MB. (**a**) Pathway enrichment analysis shows enriched pathways in MB subgroups compared to normal samples. Analysis was performed with the GSE124814 dataset. (**b**) Clustering analysis was done using the R2 genome analysis and visualization platform to examine the expression patterns of autophagy-related genes across four distinct subgroups of MBs (GSE85217). (**c**) Box plot showing *mRNA* expression of *REST* in SHH-MB patients (GSE85217) categorized as high REST (HR) and low REST (LR) samples. (**d**) Heatmap shows the clustering of HR and LR SHH-MB samples based on the expression of autophagy-related genes in GSE85217.

Transcriptomic analyses of human MB tumors (GSE124814) validated the above findings and also revealed a significant differential enrichment in pathways associated with chromatin remodeling and organization, as well as neurogenesis and cell cycle regulation in all molecular subgroups compared to normal cerebella (Fig. 1a). Notably, except for group 3, all other MB subgroups demonstrated significant enrichment in autophagy-related pathways compared to normal samples (Fig. 1a). To better understand the expression profile of autophagy-related genes within MB subgroups, we performed unsupervised clustering analysis using the microarray dataset (GSE85217) and the RNA-seq dataset (GSE148389). These studies revealed distinct differential expression profiles of autophagy-related genes between the MB subgroups (Figs. 1b and S3). As seen in Fig. 1b, SHH- and WNT-MBs were segregated into two unique clusters and separated by group 3 and group 4 samples, which appeared to have a more similar gene expression pattern. Of note, the top-upregulated genes in SHH tumors showed decreased expression in the adjacent group 3 and group 4 samples in the GSE85217 dataset (Fig. 1b). The RNA-seq dataset (GSE148389) yielded similar clustering results, reinforcing the observation that SHH MBs exhibit a unique expression profile of autophagy-related genes, distinct from group 3 and group 4 subgroup tumors (Fig. S3). These findings provide evidence that the autophagy-related regulome is different between the SHH subgroup and non-SHH MB subgroups.

### REST elevation in SHH-MB samples promotes autophagy

REST is a transcriptional repressor and is important for the survival of neurons under conditions of cellular stress^35,36^. Its expression is elevated in human SHH MB tumors and is associated with poor prognosis for patients^9^. Autophagy is also important for neuronal homeostasis and survival, although its induction can also result in cell death^21,37^. To identify REST-associated changes in the expression of autophagy-related genes in SHH MBs, we divided SHH MBs in the GSE85217 dataset into high- (HR) and low-REST (LR) samples based on their *REST* gene expression and performed clustering analyses (Fig. 1c,d). As shown in Fig. 1d, HR samples clustered separately from the LR samples, and exhibited marked differences in the expression of autophagy-related genes. Analysis of an additional dataset (GSE148389) also corroborated the findings from GSE85217. Again, HR samples and LR samples could be clearly demarctaed with respect to the expression pattern of autophagy-related genes (Fig. S4). These data strongly suggest autophagy-related genes are differentially expressed in HR- and LR-SHH MB samples. It also raises the possibility that REST may control autophagy in SHH MBs.

**Figure 2:**
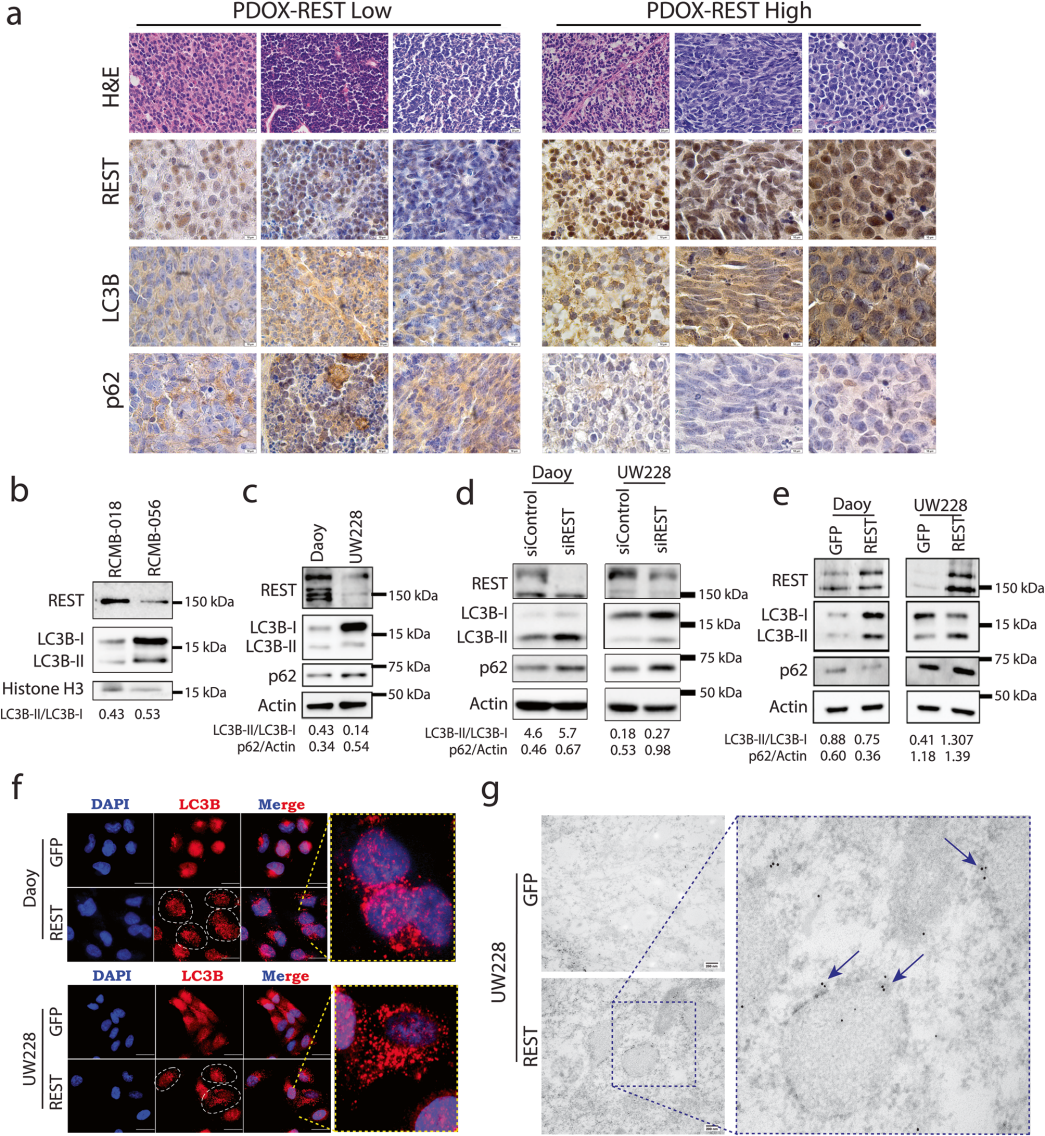
REST regulates autophagy in SHH MB. (**a**) Immunohistochemical staining of patient-derived orthotopic xenografts (PDOX) with high and low REST expression for autophagy markers, LC3B and p62. Scale bar: [20 µm for H&E and 10 µm for immunohistochemistry]. (**b**) Western blot analysis of autophagy markers in PDOX samples, RCMB-018 and RCMB-056. The densitometry ratios of LC3B-II/LC3B-I are shown below the Western blot images. Western blot of SHH-MB cell lines, Daoy and UW228 to show (**c**) basal levels of REST, LC3B-I, LC3B-II and p62 and changes in their levels following (**d**) REST knockdown and (**e**) overexpression. The densitometry ratio of LC3B-II/LC3B-I (top row) and p62/Actin (bottom row) is shown below the Western blot image. (**f**) Immunofluorescence images of parental and REST-overexpressing isogenic cells showing LC3B staining. Zoomed-in images highlight punctate staining of LC3B in REST-overexpressing cells. Scale bar: [25 µm] (**g**) Transmission Electron Microscopy (TEM) images displaying immunogold labeling of LC3B in parental and REST-overexpressing UW228 cells. The zoomed-in images show the localization of LC3B in an autophagosome (arrow), (scale bar = 200 nm (50000X)).

As a first step in experimentally defining the role of REST in autophagy, we performed immunohistochemical staining of patient-derived orthotopic xenograft (PDOXs) sections of HR and LR SHH-MB tumors. As expected, LC3B exhibited cytoplasmic staining in all samples (Fig. 2a). However, in HR SHH-MBs, LC3B staining was intense and punctate, as opposed to a more homogenous distribution in LR SHH tumors (Fig. 2a). A significant reduction in p62 was seen in HR SHH-MB PDOX sections compared to LR samples (Fig. 2a). Together, these data suggest that REST elevation in SHH-MBs is correlated with an increase in markers of autophagic flux. Western blot analyses using lysates of PDOX tumors (RCMB018 and RCMB056) and cell lines (Daoy and UW228) showed that higher REST expression was associated with reduced accumulation of LC3B-I, whereas increased accumulation of LC3B-I was observed in low-REST samples (Fig. 2b,c). REST and p62 levels were inversely correlated in Daoy and UW228 cells, which was confirmed by densitometry (Fig. 2c), suggesting a potential blockade of autophagic flux in UW228 cells with low endogenous levels of REST relative to Daoy cells. Next, *REST* knockdown was carried out in Daoy and UW228 cells using pooled *siRNA* to assess if REST controls autophagic flux. *REST* silencing promoted increased accumulation of LC3B-II in Daoy and both LC3B-I and -II in UW228 compared to control *siRNA* transduced cells (Fig. 2d). Additionally, for both cell lines, densitometry showed that *REST* knockdown caused an increase in p62 levels (Fig. 2d). Next, *REST* transgene was stably overexpressed in Daoy and UW228 cells to generate Daoy-REST and UW228-REST cells. These isogenic pairs of cells were then studied by Western blotting to demonstrate that *REST* overexpression promoted an increase in both LC3B-I and -II and decreased p62 levels in Daoy cells, as opposed to UW228 cells where LC3B-I to -II conversion was increased, but p62 levels remained unchanged (Fig. 2e). Immunofluorescence assays revealed an increase in punctate LC3B staining in both Daoy-REST and UW228-REST cells, compared to their isogenic parental cell, raising the possibility of increased LC3B-II localization to autophagosomes (Fig. 2f). Indeed, electron microscopy and immunogold labeling confirmed an increase in LC3B accumulation (arrows) around vesicular structures, likely autophagosomes, in UW228-REST compared to UW228 cells (Fig. 2g). These data indicate that REST may also control the process of LC3B-I to LC3B-II conversion, and possibly LC3B-II degradation. The isogenic pairs of cells were also treated with the autophagy inhibitors, Bafilomycin and Hydroxychloroquine, to assess cell survival. The cytotoxicity data shown in Fig. S5 demonstrate that while both cell pairs were responsive to autophagy inhibition, REST overexpression caused a significant increase in sensitivity to the drugs. These findings indicate that autophagy is a pro-survival mechanism in these cells.

### REST-driven autophagy requires HIF1α

**Figure 3:**
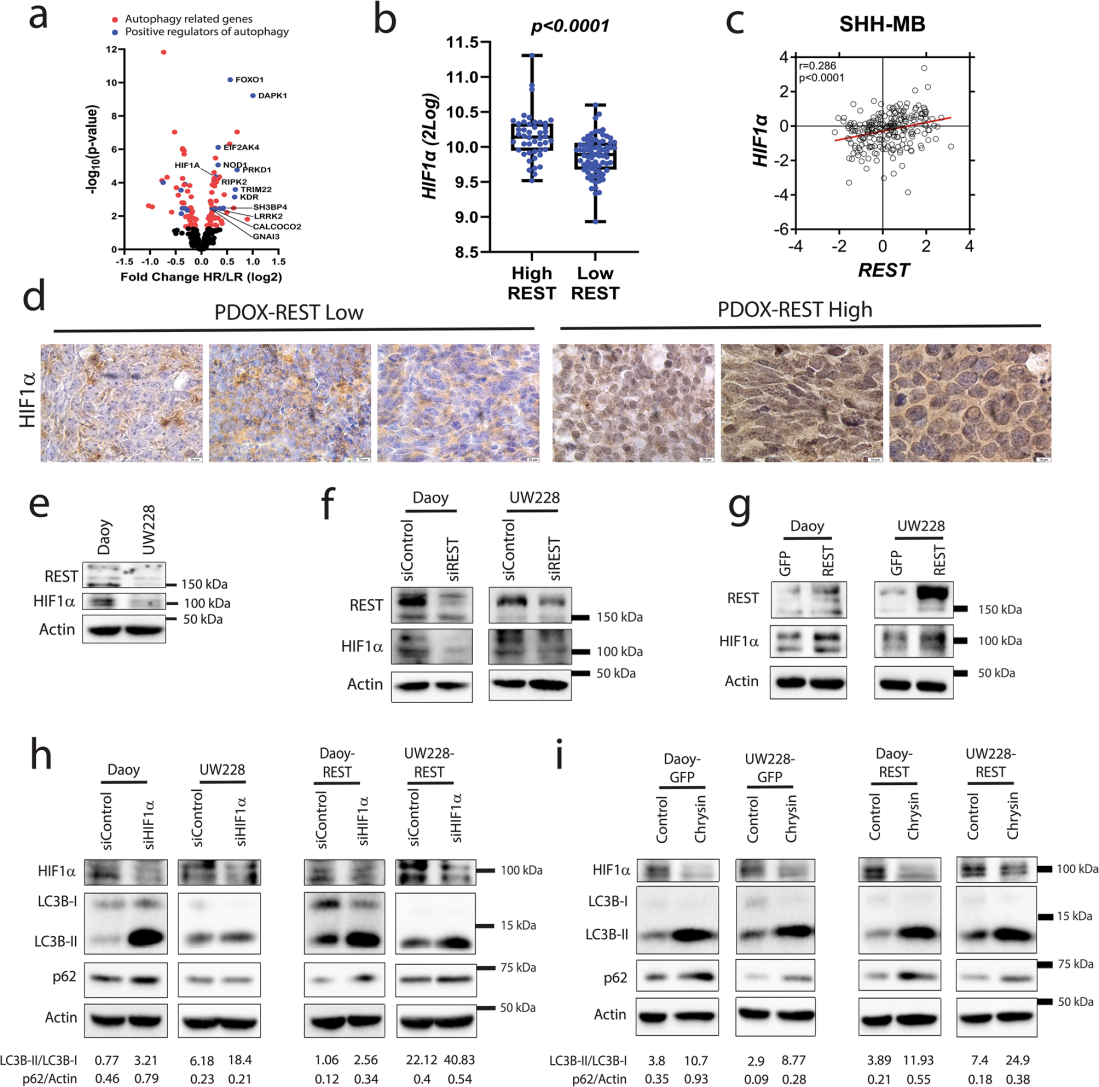
HIF1α is required for REST-dependent autophagy in SHH MBs. (**a**) Volcano plot showing the expression of positive regulators of autophagy including HIF1α in HR compared to LR SHH-MB samples (GSE85217). (**b**) Box plot showing *mRNA* expression of *HIF1α* in HR and LR SHH-MB samples (GSE85217). (**c**) Scatter plot of correlation of *REST* mRNA expression and *HIF1α mRNA* expression (GSE85217; n = 223). (**d**) Immunohistochemical staining of HIF1α in SHH-MB PDOX with high and low expression of REST. Scale bar: [20 µm]. Western blot of SHH-MB cell lines, Daoy and UW228 to show (**e**) the basal levels of REST and HIF1α, and changes in their levels following (**f**) *REST* knockdown or (**g**) overexpression. Western blot to show the change in HIF1α, LC3B-I, LC3B-II and p62 level after *siRNA* (**h**) or pharmacological (Chrysin) ablation of HIF1α in parental and (**i**) REST-overexpressing Daoy and UW228 cells. Densitometry ratios of LC3B-II/LC3B-I (top row) and p62/Actin (bottom row) are shown below the Western blot images.

Consistent with our previous data^13^, the expression of *HIF1α*, a known regulator of autophagy^34,38^, was significantly higher in HR SHH-MB samples compared to LR tumors included in the GSE85217 data set (Fig. 3a,b). Other positive regulators of autophagy, including *FOXO1, DAPK1, NOD1, EIF2AK4, PRKD1,* and *TRIM22* were also upregulated in HR SHH-MBs compared to LR samples (Fig. 3a). Since, *REST* and *HIF1α* showed a significant positive correlation in SHH-MB tumors (Fig. 3c) and our previous work showed that inhibition of REST-associated lysine-specific demethylase-1 (LSD1) caused a reduction in *HIF1α* transcript levels^13^, we asked if HIF1α is required for REST-dependent autophagy. First, IHC staining of HR- and LR- SHH MB PDOX sections revealed differences in sub-cellular localization of HIF1α in that its staining was predominantly cytoplasmic in LR-PDOX tumors and nuclear in HR-PDOX tumors (Fig. 3d). Second, Western blotting showed REST and HIF1α levels to be higher in Daoy compared to UW228 cells (Fig. 3e). Knockdown of *REST* with pooled *siRNA* caused a reduction in HIF1α levels compared to control *siRNA*-transfected cells (Fig. 3f). Also, ectopic expression of *REST* in both cell lines caused an increase in HIF1α protein levels, confirming a role for REST in modulating HIF1α protein expression (Fig. 3g).

We next studied the requirement of HIF1α for REST-dependent autophagy. HIF1α knockdown using pooled specific-*siRNA* increased the level of LC3B-II in both Daoy and UW228 cells when compared to control *siRNA* transfected control cells (Fig. 3h). An accumulation of p62 was observed only in Daoy cells but not in UW228 cells (Fig. 3h). Also, under conditions of REST overexpression, *HIF1α* knockdown resulted in the accumulation of LC3B-II and p62 for both Daoy and UW228 cells (Fig. 3h). Pharmacological degradation of HIF1α by Chrysin^39,40^ also led to the accumulation of LC3B-II and p62 in Daoy and UW228 cells (Fig. 3i). Similar results were observed in Daoy-REST and UW228-REST cells (Fig. 3i). Together these finding suggest that HIF1α loss may block the maturation of autophagosomes.

### REST modulates von Hippel-Lindau (VHL) tumor suppressor levels

**Figure 4:**
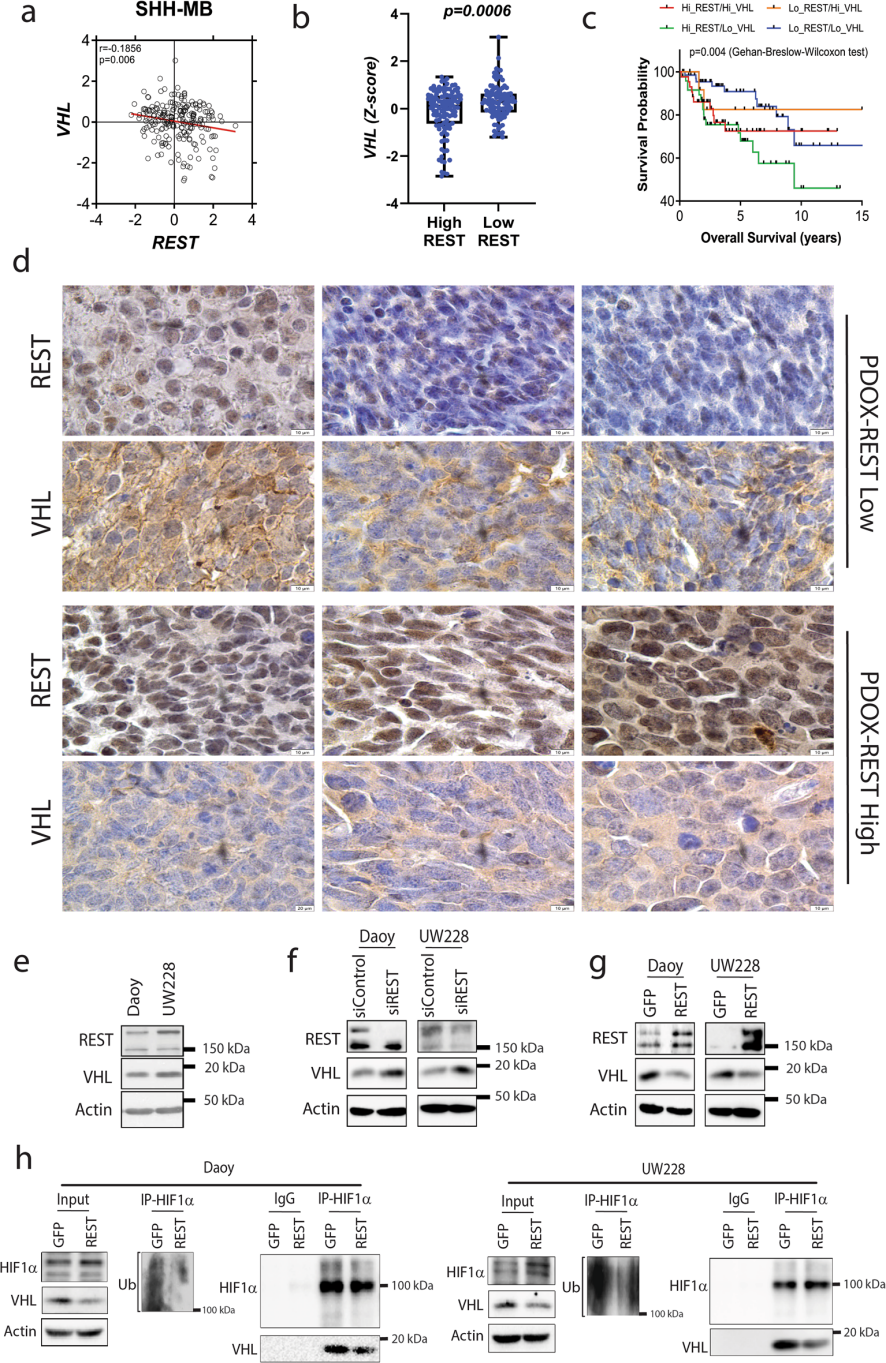
REST elevation causes a decline in VHL expression. (**a**) Scatter plot of correlation of *REST* mRNA expression and *VHL* mRNA expression (GSE85217; n = 223). (**b**) Box plot to show significant decrease (p = 0.0006) of *VHL* expression in HR samples compared to LR SHH-MBs (GSE85217). (**c**) Kaplan–Meier plot to demonstrate significant differences in overall survival of SHH-MB patients based on the relative expression of *REST* and *VHL* in the tumors (GSE85217). (**d**) Immunohistochemical staining of VHL in SHH-MB PDOX with high and low expression of REST. Scale bar: [20 µm]. (**e**) Western blot showing expression of VHL in Daoy and UW228 cell lines. Changes in VHL levels were studied by Western blotting after *siRNA*-mediated *REST* silencing (**f**) and *REST* overexpression (**g**) in Daoy and UW228 cells. (**h**) Co-immunoprecipitation of HIF1α and VHL in isogenic parental and REST overexpressing Daoy and UW228 cells. Cell lysates were subjected to immunoprecipitation with anti-HIF1α antibody followed by Western blot analysis with anti-ubiquitin (Ub) and anti-VHL antibodies.

Differences in the sub-cellular localization and levels of HIF1α in HR-and LR-SHH-PDOX tumors led us to ask if REST had a role in the post-transcriptional regulation of HIF1α. VHL is a key subunit of a multiprotein ubiquitin ligase that interacts with nuclear HIF1α and promotes its ubiquitination and transport to the cytoplasm for 26S proteasomal degradation^41^. To assess if deregulation of *VHL* contributed to REST-dependent changes in HIF1α levels, we first showed a significant negative correlation (p = 0.006) between *REST* and *VHL* in SHH MB samples from the microarray data set (GSE85217) (Fig. 4a). Also, *VHL* transcript levels were significantly lower in HR samples compared to LR samples, and patients with high-*REST* and low-*VHL* expression in their tumors exhibited the worst survival relative to patients with tumors expressing high-*REST*/high-*VHL*, low-*REST*/high-*VHL*, or low-*REST*/low-*VHL* (Fig. 4b,c). IHC analysis of SHH-MB PDOX sections also confirmed higher levels of VHL in low-REST compared to high-REST tumor sections (Fig. 4d). Expression of VHL was inversely correlated with REST levels in Daoy and UW228 cells (Fig. 4e). Daoy cells also showed an increase in a higher molecular weight form of VHL (~ 200 KD) compared to UW228 cells (Fig. S6). Further, *REST* knockdown and elevation in Daoy and UW228 cells caused upregulation and downregulation of *VHL* protein, respectively (Fig. 4f,g). Also, ectopic expression of REST was associated with an increase in slower mobility forms of VHL (~ 200KDa) compared to the isogenic parental cells, suggesting that REST elevation leads to post-translational modification of VHL, which remains to be defined (Fig. S7). Lastly, co-immunoprecipitation assays using anti-HIF1α antibody revealed reduced HIF1α ubiquitination and decreased VHL pull-down in high-REST MB cells compared to low-REST cells (Fig. 4h). These data suggest that REST elevation promotes HIF1α stabilization by preventing its VHL-mediated ubiquitination and proteasomal degradation. Collectively, we propose that REST elevation promotes HIF1α stability and autophagy in SHH MBs, which is dependent on downregulation of VHL under these conditions.

## Discussion

Cerebellar granule neuron progenitors (CGNPs), the cells of origin of SHH-MBs, give rise to cerebellar granule neurons, which comprise ~ 80% of neurons in the brain^42,43^. Developmentally, CGNPs undergo a burst of proliferation in the early postnatal period, leading to a massive expansion of the external granule layer (EGL) prior to cell cycle exit, onset of neuronal differentiation, and migration to their final destination in the internal granule layer^44^. This carefully orchestrated sequence of events is driven by SHH signaling, and its deregulation is a key event in SHH-driven MB genesis^44,45^. REST also contributes to the maintenance of proliferation by destabilizing p27 and blocking cell cycle exit, as well as by transcriptionally repressing the expression of neuronal differentiation genes^9,46^.

Importantly, this burst of GCNP proliferation occurs in the context of a hypovascularized EGL and a hypoxic microenvironment, and HIF1α expression–a transcriptional activator of genes involved in vascular remodeling, is upregulated in the early postnatal EGL^33,47^. Our previous work showed that elevated REST expression in CGNPs promotes vascular growth, although a role for HIF1α in this process remained to be demonstrated^48^. Hypoxia is also a driver of autophagy^34,49^. However, the importance of autophagy for CGNP survival during its proliferative phase in the postnatal cerebellum, as well as mechanisms underlying HIF1α upregulation in proliferating CGNPs were not defined. The current work addresses some of these gaps in SHH-MBs which originate from CGNPs. Autophagy is a cytoprotective mechanism in the brain and is important for preventing neurodegenerative diseases^16,17,50–54^. Thus, our findings that autophagy is important to the biology of a neuronal tumor such as MB, is not entirely surprising. Interestingly, we found that the profile of expression of autophagy-related genes in SHH-MBs is distinct from that in WNT, group 3, and group 4 MBs, which may reflect their different cells of origin (Figs. 1b and S3). We show that autophagy supports tumor cell survival.

REST is thought to play a protective role during aging by modulating autophagy flux and senescence in neurons^21^. In SHH MBs, higher and lower REST levels in RCMB-018/Daoy cells and RCMB-056/UW228 cells were associated with higher and lower autophagic flux, respectively. Unexpectedly, REST also appears to control the process of LC3B-I to -II conversion, which is consistent with a significantly positive correlation of *ATG3* and *REST* in SHH-MB (Fig. S8). However, while REST overexpression in Daoy and UW228 cells enhanced the conversion of LC3B-I to LC3B-II, p62 degradation was noted only in Daoy-REST cells, raising the possibility that cargo loading and autophagosome degradation may be impaired in UW228 cells. Work from other groups has shown that hypoxia induces autophagy in the tumor microenvironment, and HIF1α transcriptionally activates the expression of BCL2/adenovirus e1B 19 kDa protein-interacting protein 3 (*BNIP3*) expression^55,56^. The resultant competition with Beclin1 for interaction with Bcl2 is thought to facilitate nucleation and enhancement of autophagic flux^57^. Our data raise the possibility that HIF1α may also regulate the elongation and maturation steps of autophagy. Our work is one of the first to connect REST-HIF1α axis to autophagy in SHH-driven MBs. The REST-HIF1α connection is consistent with our previous study, where inhibition of REST-associated LSD1 activity caused a decline in HIF1α levels^13^. At this time, we cannot rule out the possibility of a direct role for REST in the transcriptional activation of *HIF1α* expression. It is important to note that while REST is described as a canonical transcriptional repressor, it is also known to activate gene expression by recruiting proteins like TET3 hydroxylase and NSD3 to the target gene loci^58^. Of note, REST is reported to regulate approximately 20% of the repressed genes during hypoxia consequent to its own increased nuclear localization^59^. These finding suggest that REST contributes to the cellular response to hypoxia.

**Figure 5:**
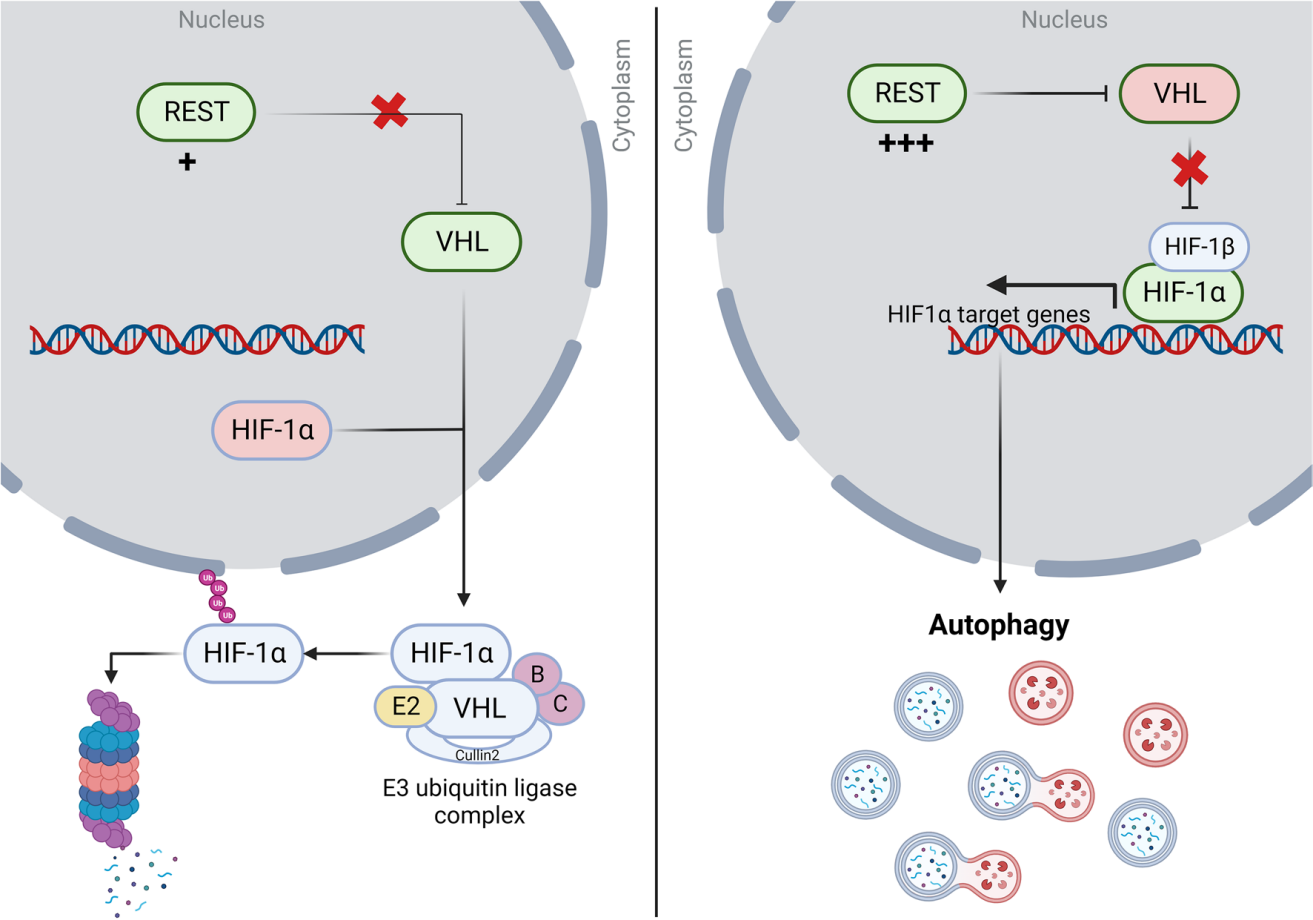
Overview of REST-mediated control of autophagy in SHH-MBs. Model figure shows REST promotes HIF1α stabilization by preventing its VHL-mediated ubiquitination and proteasomal degradation (Created with BioRender.com).

Here, we also describe a novel finding that REST elevation in SHH-MBs causes downregulation of VHL protein and consequent stabilization and nuclear retention of HIF1α (Fig. 5). Mutations in *VHL* are described in other cancers and are shown to be causal to tumor progression^60,61^. Our work suggests that REST modulates VHL protein levels in SHH-MBs. The laddering pattern of VHL expression in Fig. S7 suggests that a post-translational modification of VHL, such as ubiquitination, may be altered in a REST-dependent manner. Consistent with this possibility, VHL protein levels have been shown to be controlled by ubiquitination^62^. Whether a similar mechanism is operational in REST-driven MBs will be investigated in future studies.

Downregulation of *VHL* in SHH-MBs and its significant correlation with poor patient survival is consistent with its tumor suppressive function in other cancers such as colorectal adenocarcinoma, renal cell carcinoma, and hemangioblastomas^63,64^. REST is a driver of metastasis in SHH-MBs, and downregulation of *VHL* in these cells would cause a further worsening of survival, which is indeed the case in our high-*REST*/low-*VHL* patient cohorts. REST is also known to activate AKT signaling^9^. AKT signaling can upregulate HIF1α expression in cancers^65–67^. Thus, AKT activation may account for the decreased survival of patients with SHH MBs exhibiting high-*REST*/low-*VHL* expression. Reduction in mTORC1 activity under conditions of nutritional and other stressors is a major promoter of autophagy^68–70^. However, a report on diabetes-induced neuronal senescence found that REST elevation under these conditions was associated with a decline in mTOR-mediated autophagy^20^. HIF1α and autophagy are shown to contribute to resistance to chemotherapy and radiation in many cancers^34,49^. Given the involvement of REST in metastasis, it would be important to ask if autophagy plays a role in the therapeutic sensitivity of REST-high metastatic SHH-MBs and if treatment response can be augmented by targeting autophagy, HIF1α, or the REST-complex.

In summary, we show here that autophagy is dysregulated in MB tumors and that the molecules engaged in effecting autophagy may be subtype-specific. We have identified a novel role for REST in modulating VHL and HIF1α protein levels as well as HIF1α sub-cellular localization in SHH-MBs. Induction of autophagy, potentially in a metastatic subpopulation of SHH-MBs, may have therapeutic implications, which warrants further investigation.

### Supplementary Information

Supplementary Information 1.

Supplementary Information 2.

## Data Availability

All transcriptomic analyses were performed using previously published datasets: GSE148389 (https://www.ncbi.nlm.nih.gov/geo/query/acc.cgi?acc=GSE148389) GSE202043 (https://www.ncbi.nlm.nih.gov/geo/query/acc.cgi?acc=GSE202043) GSE124814 (https://www.ncbi.nlm.nih.gov/geo/query/acc.cgi?acc=GSE124814) GSE85217 (https://www.ncbi.nlm.nih.gov/geo/query/acc.cgi?acc=GSE85217).
