# Supplementary material for: REST-dependent downregulation of von Hippel-Lindau tumor suppressor promotes autophagy in SHH-medulloblastoma
Source: Sci Rep. 2024 Jun 12;14:13596. doi: 10.1038/s41598-024-63371-7 (PMC11169471; doi:10.1038/s41598-024-63371-7)

**REST-dependent downregulation of von Hippel-Lindau tumor suppressor promotes autophagy in SHH-medulloblastoma.**

Ashutosh Singh^1^, Donghang Cheng^1^, Jyothishmathi Swaminathan^1^, Yanwen Yang^1^, Yan Zheng^1^, Nancy Gordon^1^ and Vidya Gopalakrishnan^1,2,3,4*^.

^1^Department of Pediatrics Research, The University of Texas MD Anderson Cancer Center, 1515 Holcombe Blvd, Houston-Unit 853, Texas 77030.

^2^Center for Cancer Epigenetics, The University of Texas MD Anderson Cancer Center, 1515 Holcombe Blvd, Houston-Unit 853, Texas 77030.

^3^Brain Tumor Center, The University of Texas MD Anderson Cancer Center, 1515 Holcombe Blvd, Houston-Unit 853, Texas 77030.

^4^The University of Texas MD Anderson Cancer Center and UTHealth Graduate School for Biomedical Sciences, 1515 Holcombe Blvd, Houston-Unit 853, Texas 77030.

***Corresponding author:**

Vidya Gopalakrishnan, PhD

Email: vgopalak@mdanderson.org


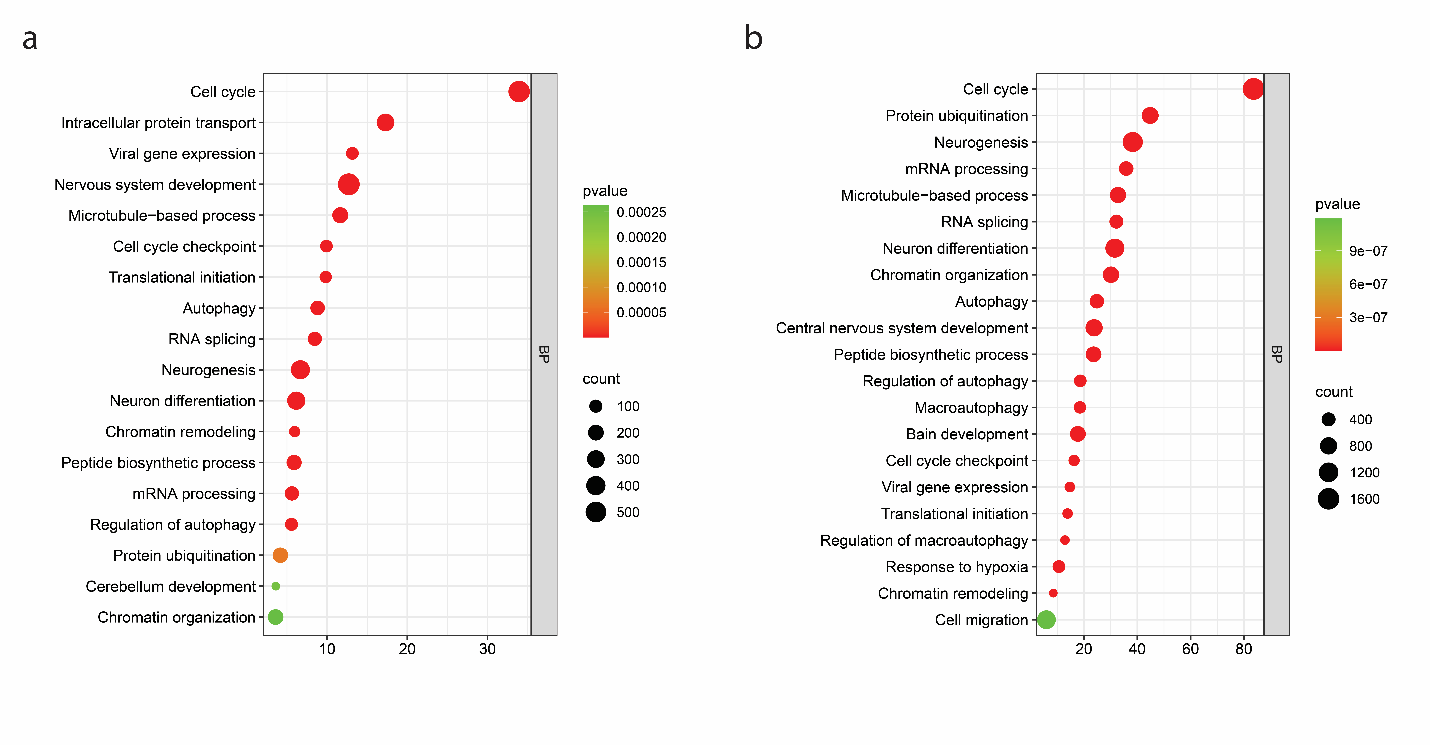
Figure S1: Pathway enrichment analysis showing enriched pathways in MB tumors relative to normal samples. Microarray (GSE202043) (a) and RNAseq (GSE148389) (b) datasets were used for this analysis.


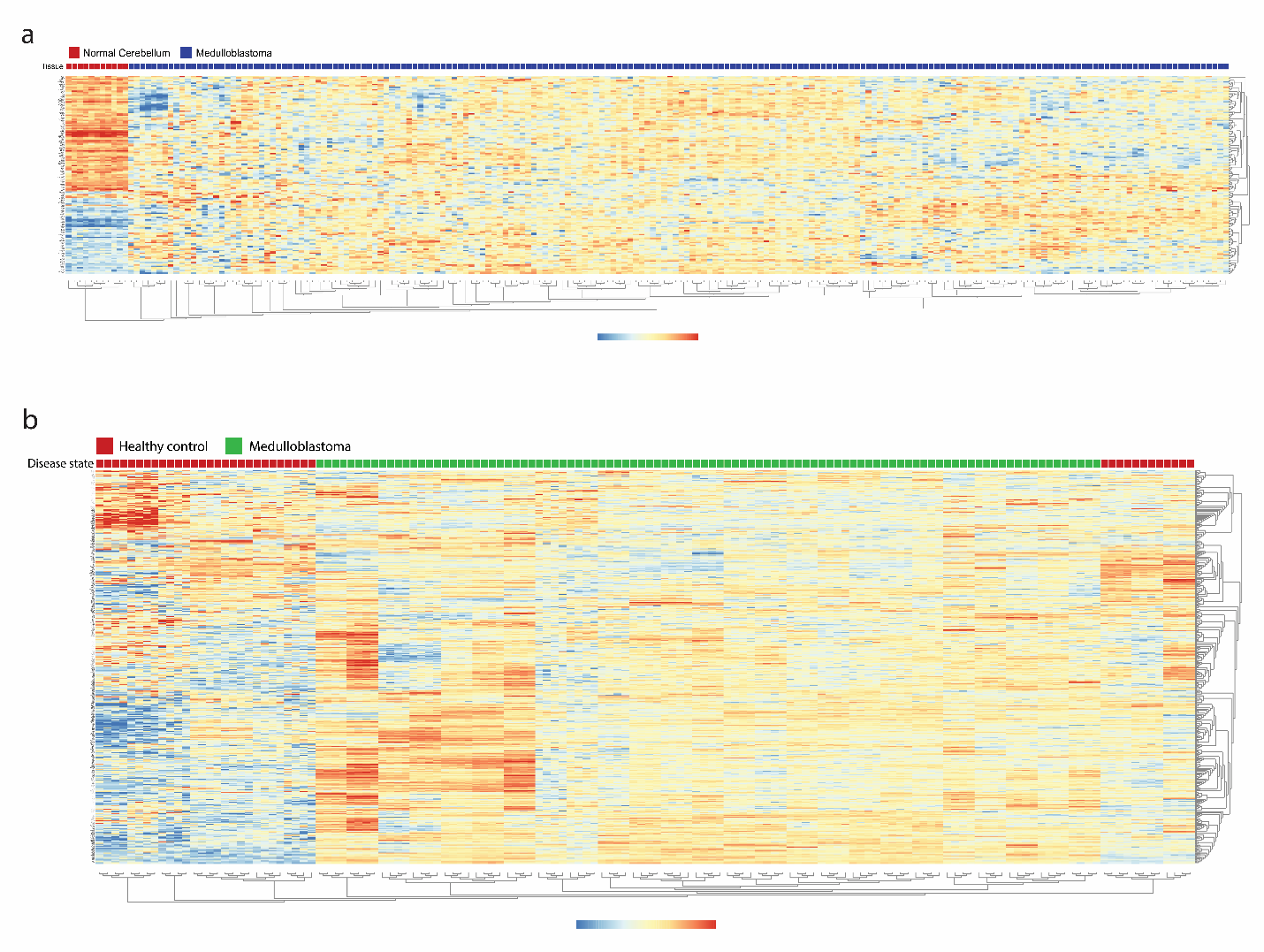
 Figure S2: Heatmap shows the clustering of MB and normal samples based on the expression of autophagy-related genes from dataset, GSE202043 (a) and GSE148389 (b).


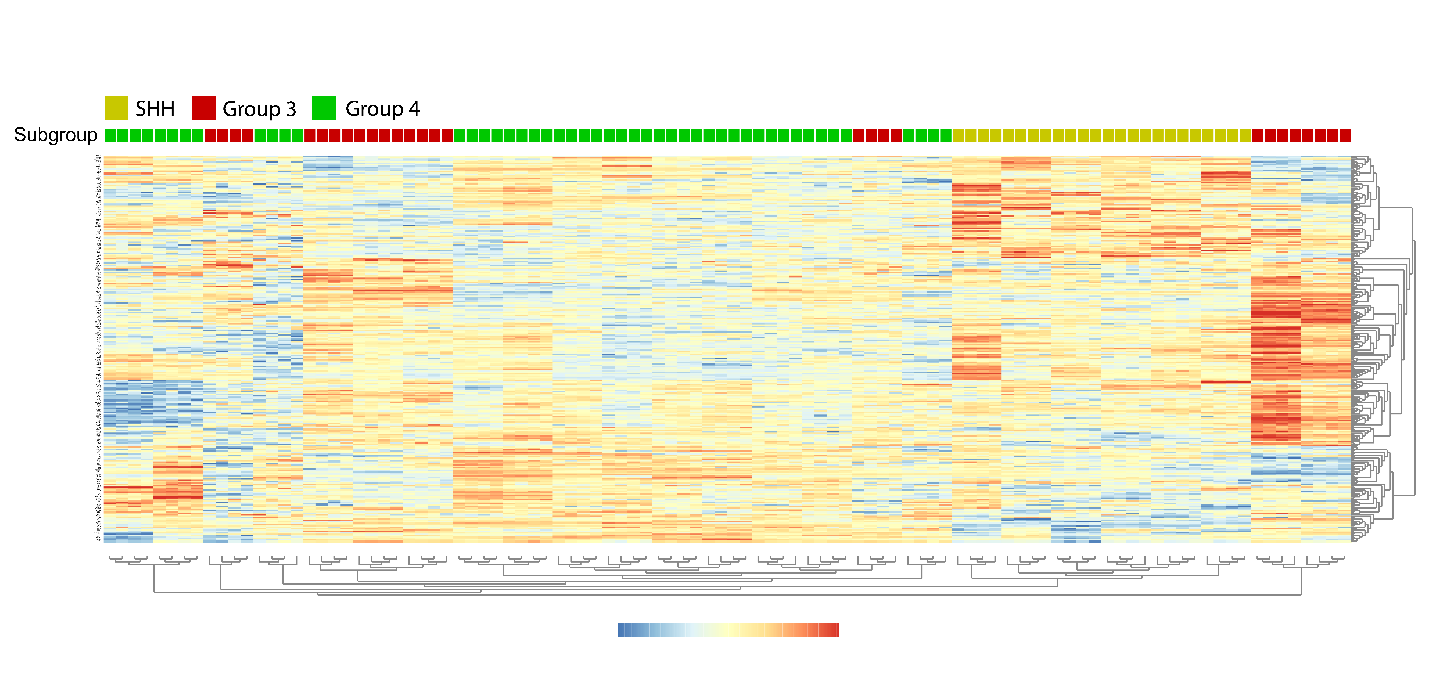


Figure S3: Heatmap shows the clustering of three MB subgroups, SHH, group 3 and group 4 based on the expression of autophagy-related genes (GSE148389).


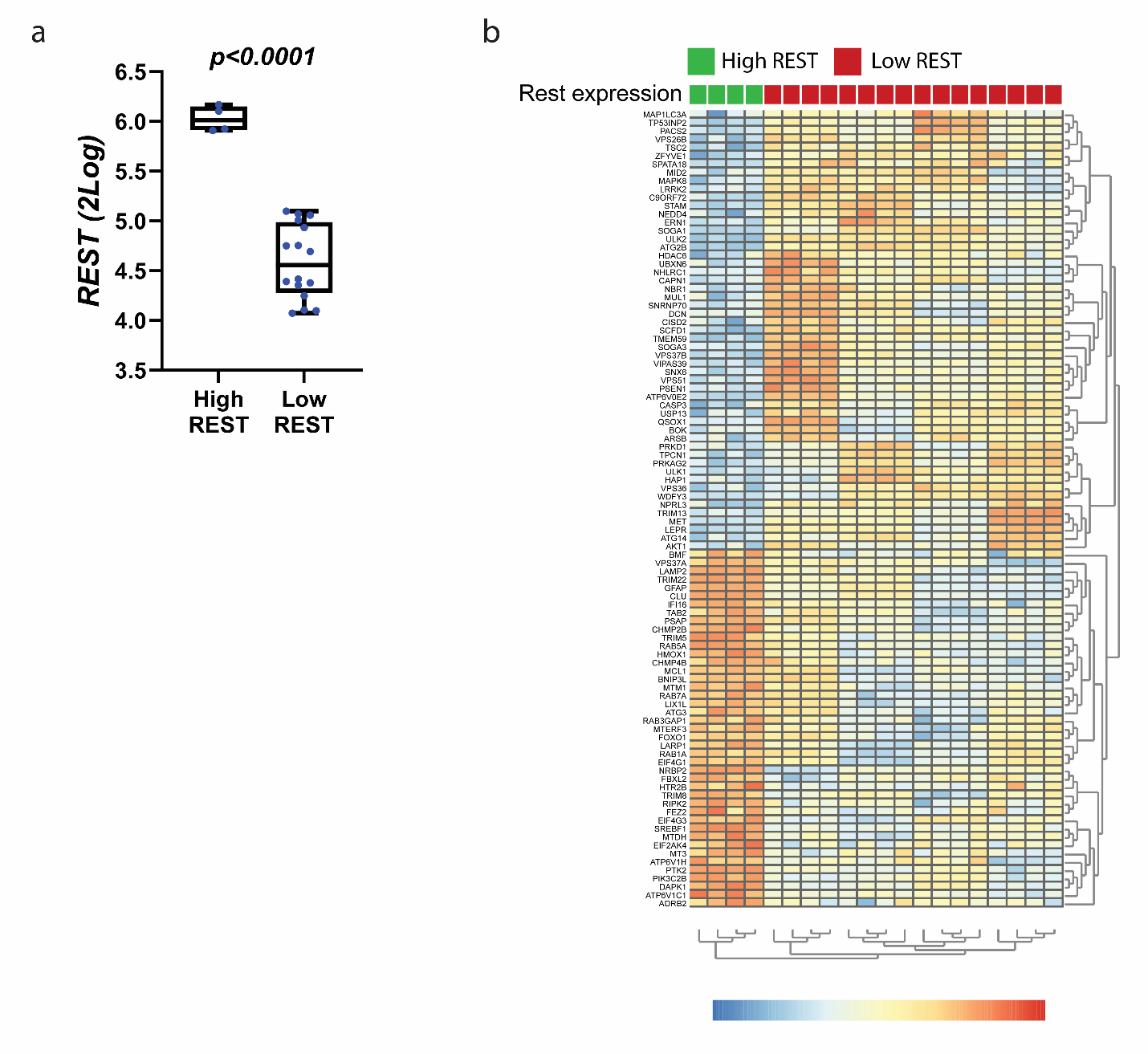


Figure S4: (a) Box plot showing *REST* *mRNA* expression in SHH MBs categorized as high REST (HR) and low REST (LR) samples in GSE148389 dataset. (b) Heatmap shows the clustering of HR and LR SHH-MB samples based on the expression of autophagy-related genes (GSE148389).


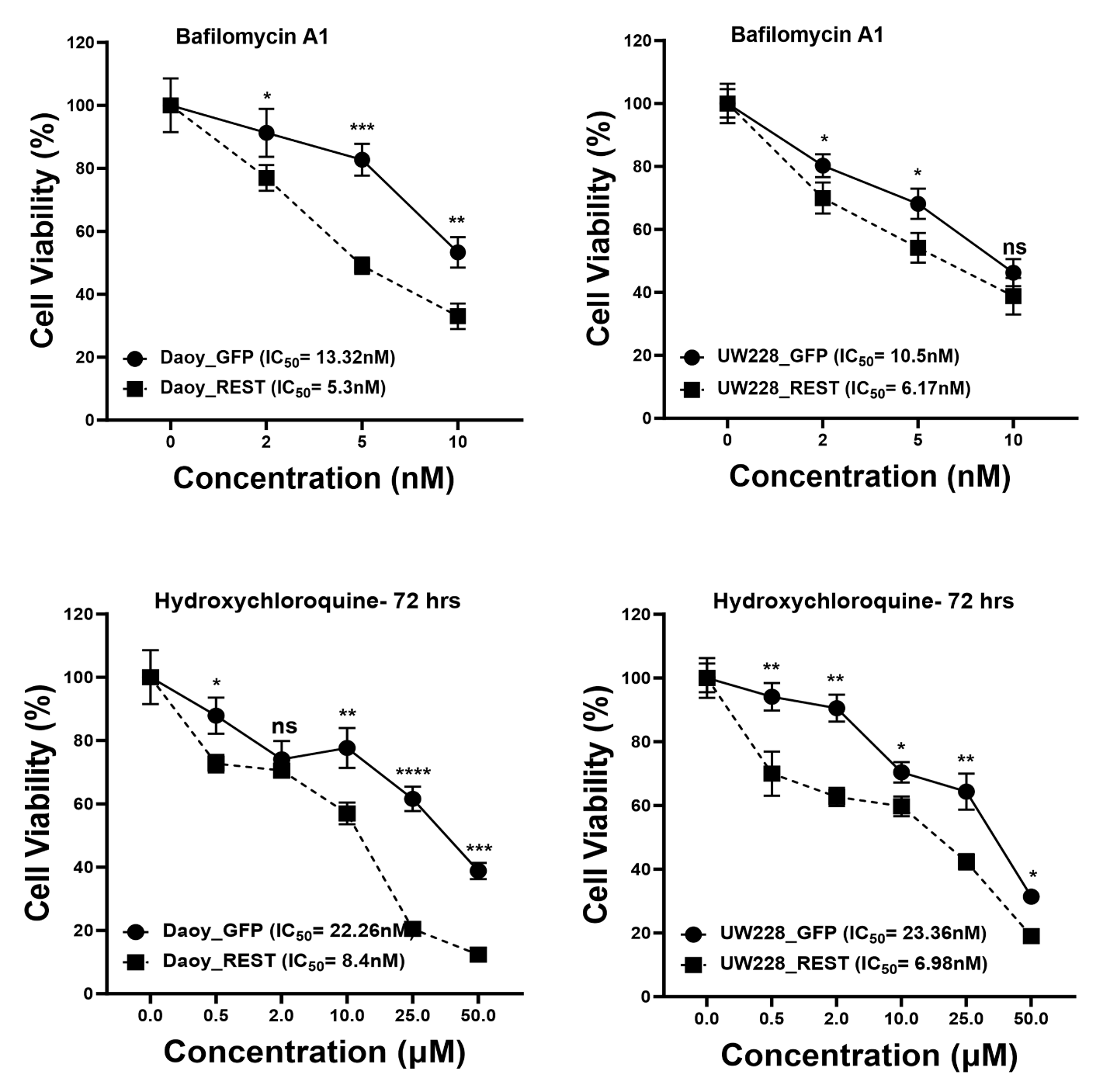


Figure S5: Cytotoxicity profile of Bafilomycin and Hydroxychloroquine against isogenic parental and REST overexpressing DAOY and UW228 cells. (p-value versus isogenic parental cells (GFP). *p < 0.05 **p < 0.01, ***p < 0.001, ****p < 0.0001, ns= not significant).


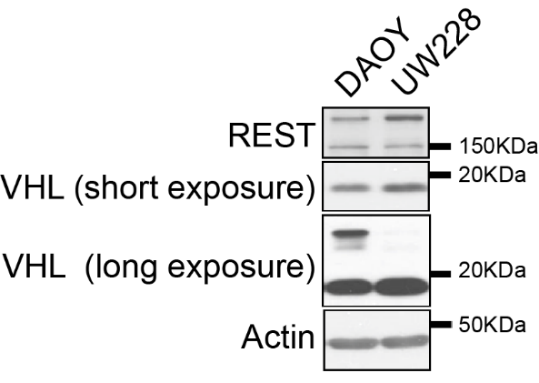


Figure S6: Western blot showing VHL expression in MB cell lines, DAOY and UW228, and a slower migrating band at ~200KD in DAOY cells.


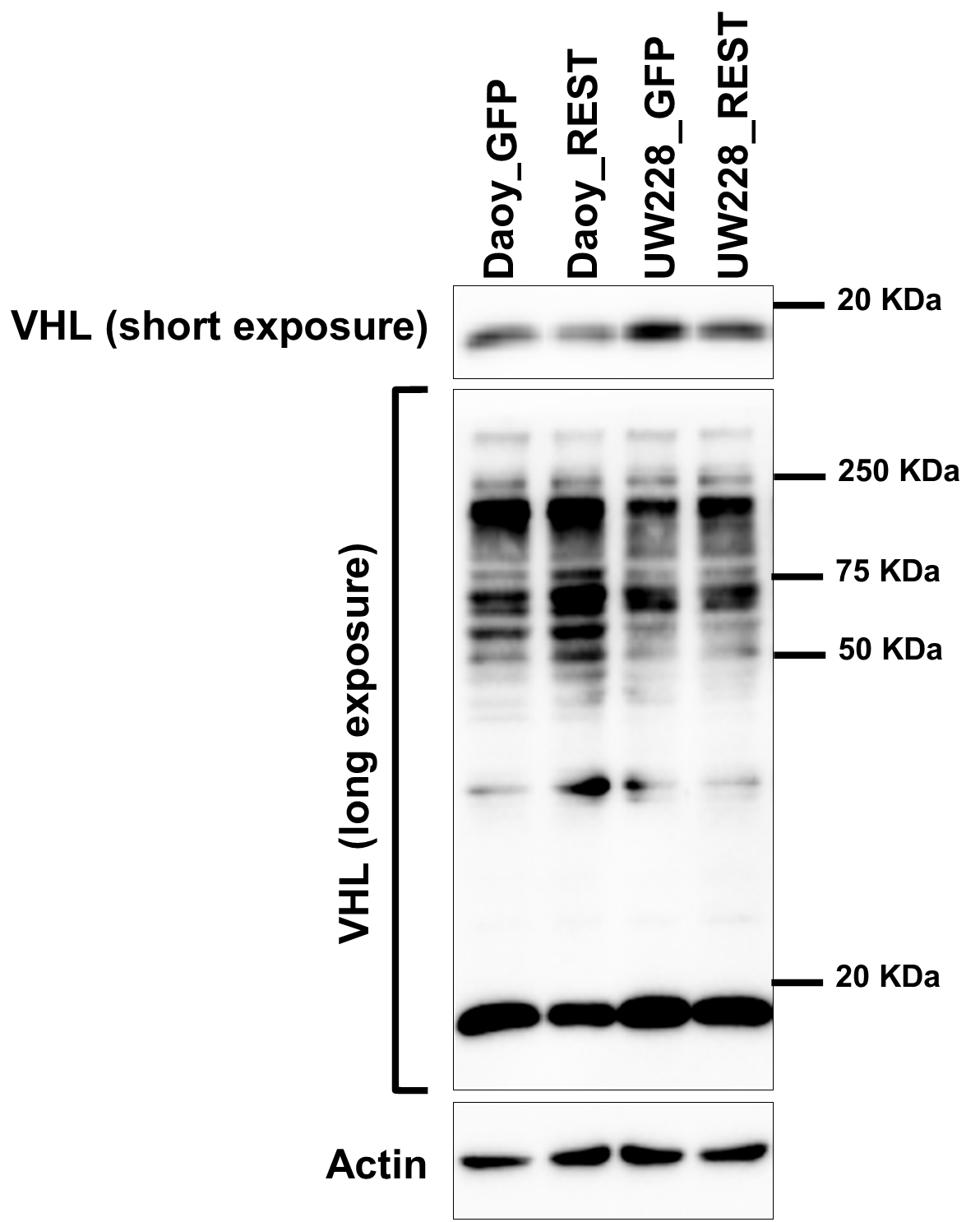


Figure S7: Western blot for VHL expression in REST overexpressing and isogenic parental MB cell lines. Low exposure and high exposure images of VHL are shown to highlight the differences in the 16 KD protein band in isogenic low and high-REST MB cells and to demonstrate changes in the laddering pattern of the protein, respectively.


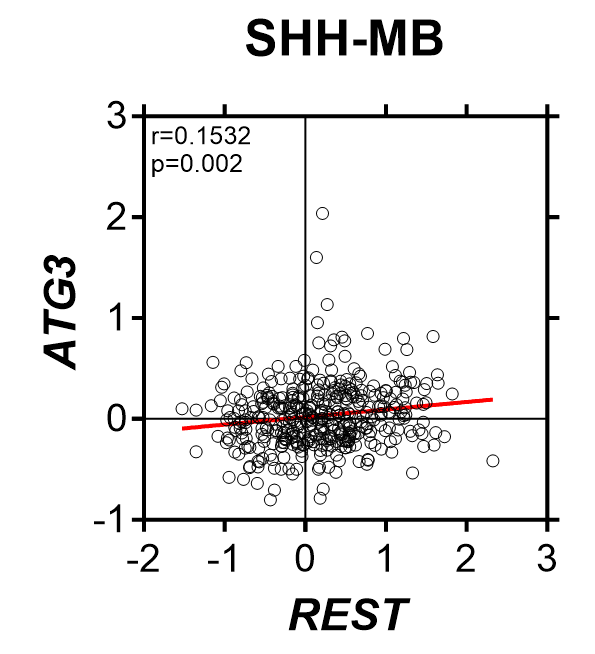


Figure S8: Scatter plot of correlation of *REST* and *ATG3* *mRNA* expression (GSE124814; n=405).

Figure S9: Uncropped scanned images shown in the indicated figures.


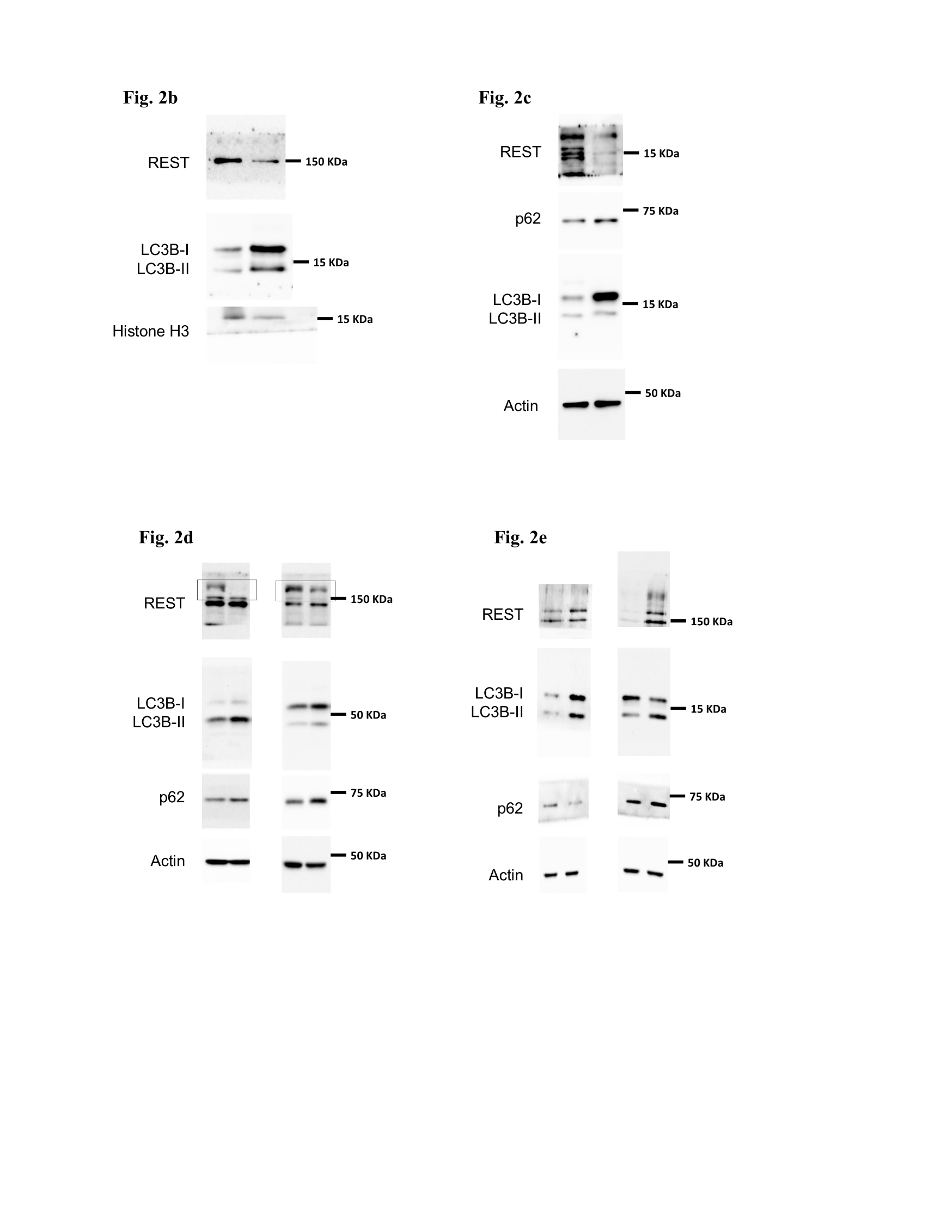


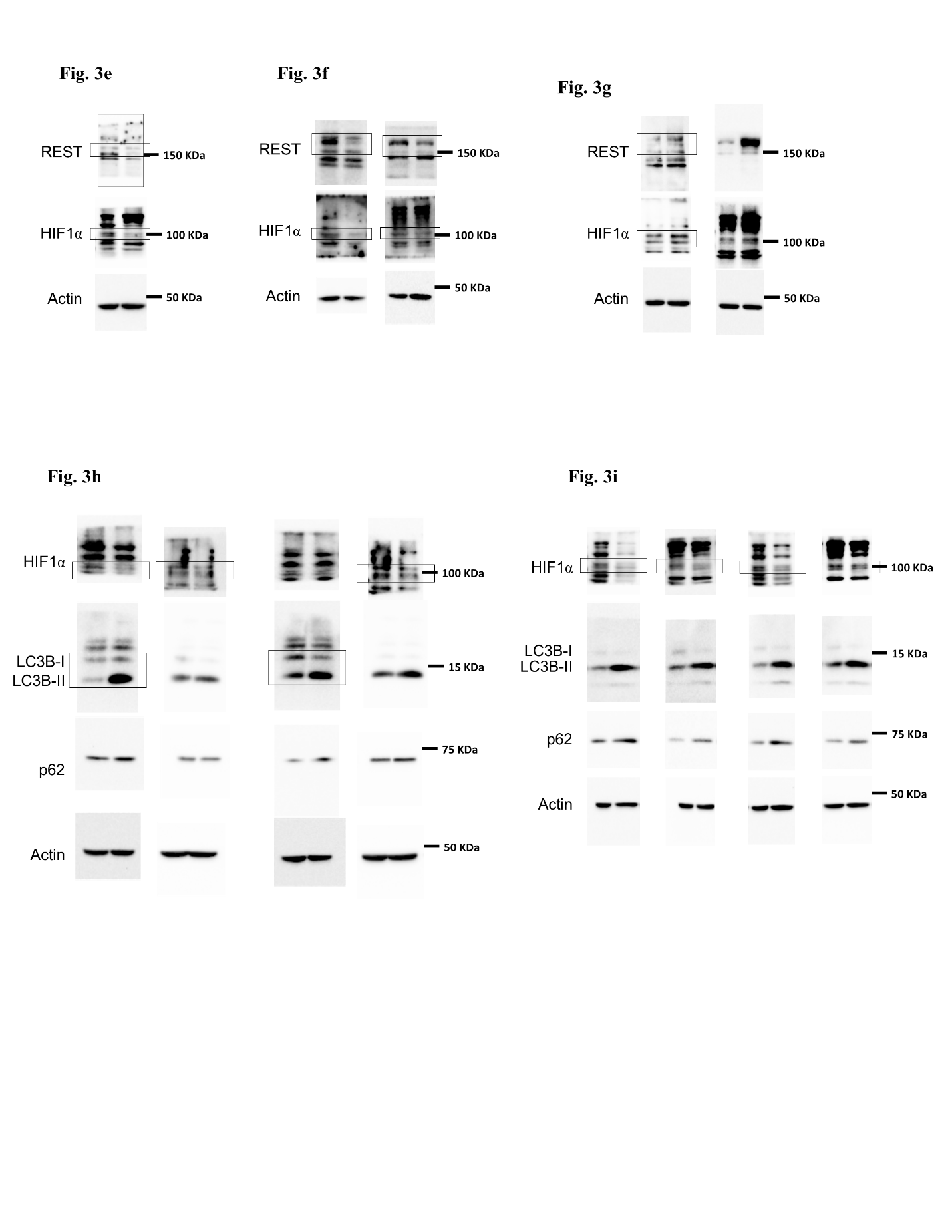


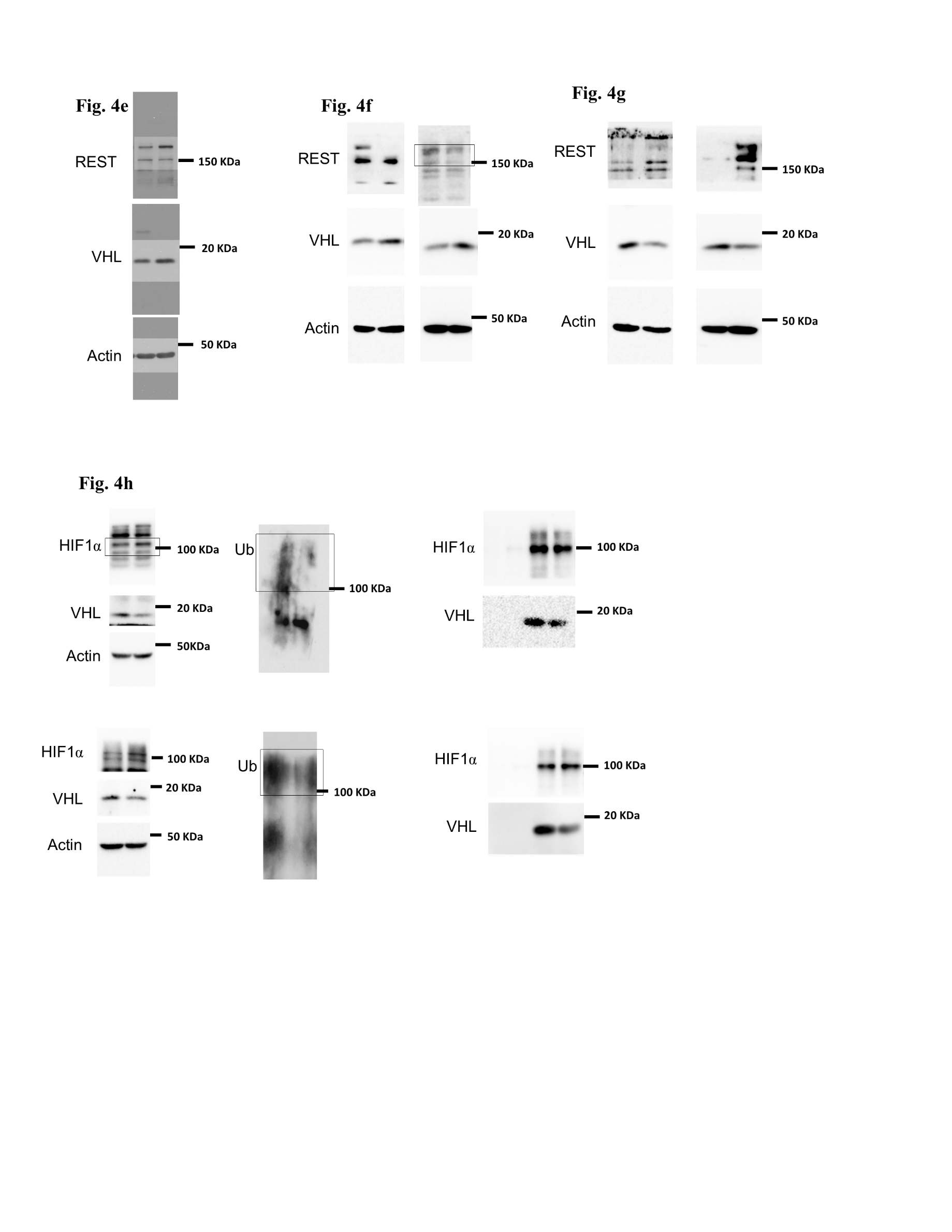

Supplement: Supplementary file 1 — Supplementary Information 1. [file 41598_2024_63371_MOESM1_ESM.docx]
